# Untangling the model muddle: Empirical tumour growth in Tasmanian devil facial tumour disease

**DOI:** 10.1038/s41598-017-06166-3

**Published:** 2017-07-24

**Authors:** Rodrigo K. Hamede, Nicholas J. Beeton, Scott Carver, Menna E. Jones

**Affiliations:** 1 School of Biological Sciences, University of Tasmania, Private Bag 55, Hobart, Tasmania 7001 Australia; 2 Centre for Integrative Ecology, Deakin University, Waurn Ponds, Victoria, 3216 Australia; 3 School of Physical Sciences, University of Tasmania, Private Bag 37, Hobart, Tasmania 7001 Australia

## Abstract

A pressing and unresolved topic in cancer research is how tumours grow in the absence of treatment. Despite advances in cancer biology, therapeutic and diagnostic technologies, there is limited knowledge regarding the fundamental growth and developmental patterns in solid tumours. In this ten year study, we estimated growth curves in Tasmanian devil facial tumours, a clonal transmissible cancer, in males and females with two different karyotypes (diploid, tetraploid) and facial locations (mucosal, dermal), using established differential equation models and model selection. Logistic growth was the most parsimonious model for diploid, tetraploid and mucosal tumours, with less model certainty for dermal tumours. Estimates of daily proportional tumour growth rate per day (95% Bayesian CIs) varied with ploidy and location [diploid 0.016 (0.014–0.020), tetraploid 0.026 (0.020–0.033), mucosal 0.013 (0.011–0.015), dermal 0.020 (0.016–0.024)]. Final tumour size (cm^3^) also varied, particularly the upper credible interval owing to host mortality as tumours approached maximum volume [diploid 364 (136–2,475), tetraploid 172 (100–305), dermal 226 (134–471)]. To our knowledge, these are the first empirical estimates of tumour growth in the absence of treatment in a wild population. Through this animal-cancer system our findings may enhance understanding of how tumour properties interact with growth dynamics in other types of cancer.

## Introduction

The propagation of cells in neoplastic diseases is driven by deregulation of control in cell proliferation, as well as several of the energetic and metabolic steps necessary to fuel cell growth and division^1, 2^. Furthermore, the control of tumour development and growth rate depends on dynamic interactions and feedback processes with the host immune system, cell-mediated mechanisms and molecular evolutionary events^3–5^. Thus, understanding the mechanistic dynamics of the tumour and the host and how they influence tumour growth is a major challenge. Mathematical models of tumour growth rates have been mostly focused on understanding the quantitative theory of increase in tumour volume^2, 6^, tumour response to radiation therapies and modelling the growth and evolution of subpopulations of cells^7–9^. In a recent study, Gerlee^10^ reviewed the historical development of tumour growth laws, from the early view of exponential growth to more complex models that take into account structural and biological attributes of tumour cells. However, most studies have been based on quantitative theory and have not used empirical data on tumour growth rates. Providing empirical data from naturally occurring neoplastic diseases in the absence of treatment has been until now a difficult task. In that sense, cancers in wildlife can be regarded as useful study systems to understand mechanistic and quantitative attributes of tumour cells. Wildlife cancers, and particularly transmissible cancers, have been increasingly recognised as a new threat to conservation and biodiversity^11, 12^. In most cancers the tumour dies and disappears within its hosts. However, in transmissible cancers the tumour cells are the infectious agent and are transmitted as a clonal cell line across hosts. Studying the developmental progression of tumours in transmissible cancers provides a unique opportunity for understanding growth rate theory and has useful applications for understanding tumour development in human and non-human cancers.

Evaluating the ecological significance of oncogenic phenomena, their epidemiology and prevalence in the wild is a challenging task^11^. This is because most cancers in wildlife are difficult to study as their clinical signs are usually imperceptible (e.g. internal tumours and therefore detected only in post mortem examinations) and in many occasions affected animals die unseen or are preyed upon before clinical signs appear^11^. The emerging view of cancers, from an evolutionary and ecological perspective, is that they are a collection of heterogeneous cells that evolve in tumour microenvironments with complex ecological processes^1, 5, 13^. Hence, evolutionary theory and mathematical modelling are becoming important tools to understand the ecology of cancers and factors associated with the stimulus and suppression of tumour growth rates^14, 15^. Establishing tumour growth curves for transmissible cancers in wildlife may help to determine optimal time periods for transmission, predict disease progression and survival after infection. In addition, assessing tumour growth dynamics and cancer progression may be used in more complex models for estimating the likelihood of metastasis.

Tasmanian devil facial tumour disease (DFTD) is a rare transmissible cancer threatening its unique host, the Tasmanian devil (*Sarcophilus harrisii*)^16, 17^. The tumour has a neuroendocrine origin^18^ and is transmitted as a clonal cell-line by direct inoculation of live tumour cells when animals bite each other^19, 20^. Since its emergence two decades ago, DFTD has evolved into several karyotypic sublineages^21^ and new evidence suggests that diploid tumours are associated with higher transmission rate, prevalence and population effects than tetraploid tumours^22^. Tetraploidy has been found to reduce cell proliferation and tumour growth rates^23, 24^. Devil facial tumours provide a unique opportunity to empirically evaluate tumour growth theory in the wild. Unlike most cancers, DFTD is not subject to treatment so repeat measures can be made across differing ploidy and facial locations. Furthermore, understanding how tumour growth rates vary between different karyotypic lineages may provide a framework to scale results from experiments in model organisms up to humans, as well as providing realistic growth trajectories to parameterise numerical models.

In this study, we provide the first empirical estimates for growth rates of diploid and tetraploid Tasmanian devil facial tumours in a natural and untreated population, and fit the data to numerical models to identify the most parsimonious growth functions. We discuss the implications of our results for calculating growth dynamics in solid tumours and for understanding the role of tumour karyotype ﻿in growth rates. We contextualise our findings for the epidemiology and effects of DFTD in wild devil populations and with application to tumour growth rate theory.

## Results

**Figure 1:**
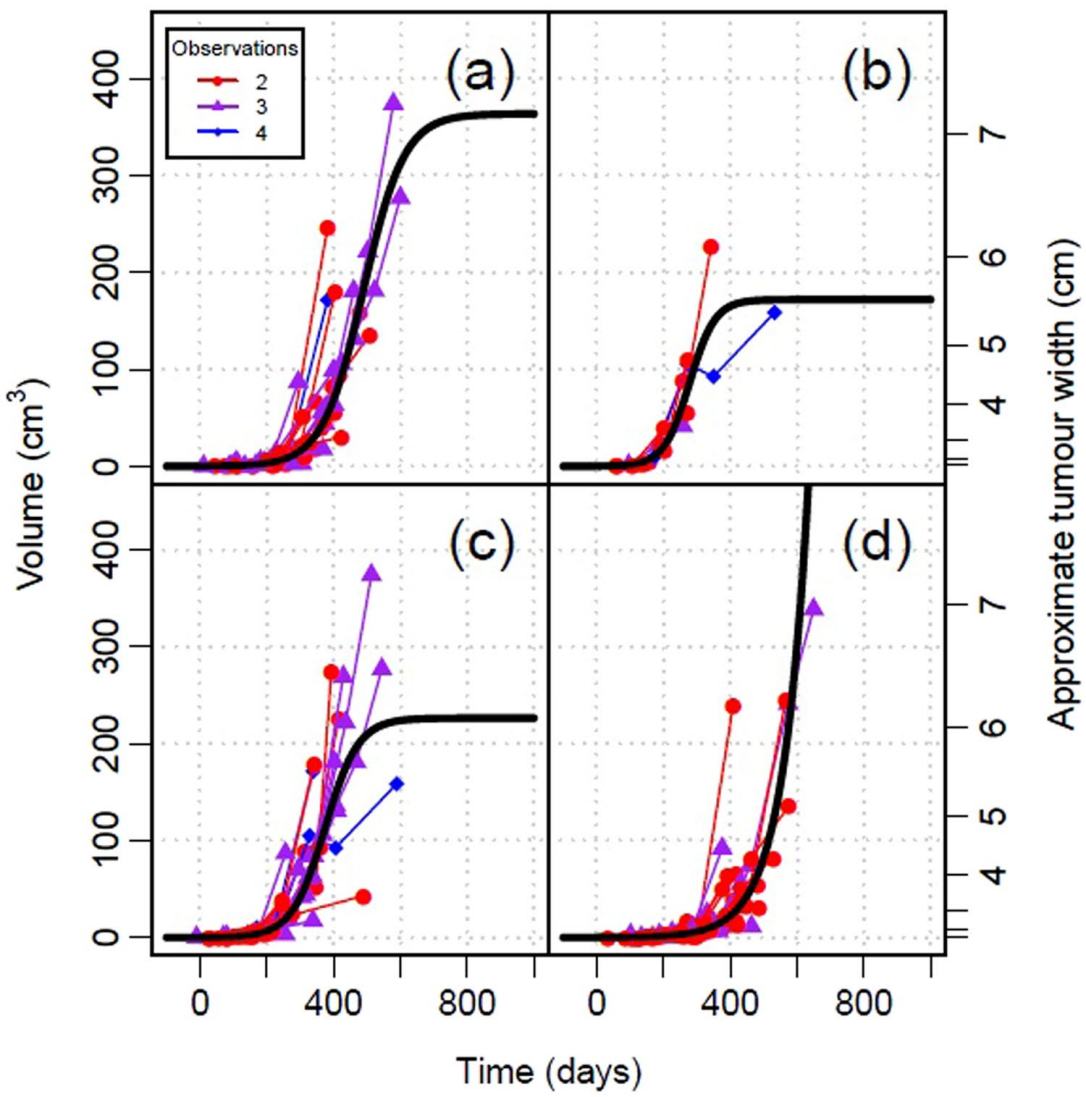
Best-fit maximum likelihood models for given tumour characteristics: (**a**) diploid, (**b**) tetraploid, (**c**) dermal and (**d**) mucosal. Volume versus time line plots for each tumour are overlaid in relation to the predicted growth curve based on their maximum-likelihood initial conditions. On each plot, time 0 represents the point at which the tumour reaches 0.125 cm^3^ in volume.

A total of 62 individual devils were captured with DFTD and a total of 87 repeat tumour measurements were recorded between 2006 and 2015 (diploid n = 32, tetraploid n = 10, unknown ploidy n = 45, dermal n = 31, mucosal n = 44, unknown location n = 12). Tumours with no karyotype data available were regarded as unknown ploidy and tumours that were located between both dermal and mucosal tissues were regarded as unknown location. We only used data for which tumour ploidy was known, regardless of location, to model tetraploid vs diploid growth rates, and combined all data for which tumour location was available, regardless of ploidy, to test the effect of tumour location (dermal vs mucosal) on growth rates. Figure 1 shows the best fit maximum likelihood models for diploid, tetraploid dermal and mucosal tumours. Table 1 suggests that the logistic model is the most effective at representing the data – it has the lowest AICc value for all data splits with the exception of dermal tumours. Although there is variation in growth rates between tumours, the tumour growth model is able to capture the increase in tumour volume over time. Estimates of tumour growth rate per day (*r*) varied with ploidy and location [diploid 0.016 (0.014–0.020), tetraploid 0.026 (0.020–0.033), mucosal 0.013 (0.011–0.015), dermal 0.020 (0.016–0.024)] but not significantly with sex [female 0.015 (0.013–0.019), male 0.016 (0.013–0.019)] (Table 2). Estimates of final tumour size (*K*, cm^3^) also varied (Table 2), though not significantly, and the upper credible interval of size varied widely owing to devil mortality as tumours approached maximum volume [diploid 364 (136–2,475), tetraploid 172 (100–305), dermal 226 (134–471), female 361 (134–1,320), male 390 (144–2,640)]. The best supported model for mucosal tumour was exponential, therefore final tumour size estimates could not be assessed.

**Table 1:** ΔAICc values for each maximum likelihood statistical model tested on tumour growth models.

		*All data*	*Diploid*	*Tetraploid*	*Mucosal*	*Dermal*	*Female*	*Male*
***Model type***	*k*	*ΔAIC values*						
Logistic	2	**0 (417.08)**	**0 (174.57)**	**0 (23.75)**	**0 (142.79)**	0.21	**0 (290.37)**	**0 (130.00)**
Exponential	1	9.05	1.56	22.94	15.42	**0 (207.30)**	5.17	1.61
Mendelsohn	2	9.77	3.04	15.26	13.12	0.86	6.76	2.43
Gompertz	2	9.18	2.87	10.23	10.61	0.43	6.56	2.15
von Bertalanffy	2	32.85	13.52	15.66	21.19	3.37	22.28	11.23

AICc values of the best fit model for each tumour characteristic are given in parentheses.

**Table 2:** Parameter estimates for growth rates per day (r) and maximum tumour volume (K) for each sex, ploidy and location. Numbers in brackets are central 95% Bayesian credible intervals.

Group	r	K
*Female*	0.015 (0.013–0.019)	361 (134–1,320)
*Male*	0.016 (0.013–0.019)	390 (144–2,640)
*Diploid*	0.016 (0.014–0.020)	364 (136–2,475)
*Tetraploid*	0.026 (0.020–0.033)	172 (100–305)
*Mucosal*	0.013 (0.011–0.015)	n/a
*Dermal*	0.020 (0.016–0.024)	226 (134–471)

## Discussion

To understand the effects of cancer treatments on tumour growth dynamics, it is essential to assess patterns of tumour growth and to mechanistically model them in the absence of treatment. Our study is, to the best of our knowledge, the first attempt to determine realistic patterns of tumour growth and mechanistically model growth in a natural and untreated population based on empirical evidence. This result is possible due to the combination of a long-term detailed dataset (replicate individuals with repeat tumour measurements) and a flexible modelling approach. We identified variation in growth rates and potential carrying capacities (the expected maximum volume) across the four distinct tumour characteristics in both males and females. A logistic model of tumour growth was the best supported in our study system for tetraploid, diploid and mucosal tumours. Exponential growth was the most supported model for dermal tumours. This suggests that for tumours outside of the oral cavity where growth is not limited by physical constraints such as bones and tissue, as is the case of tumours inside the oral cavity, there is no threshold for tumour growth within the life expectancy of infected devils.

Our findings may extend to tumour growth in other organisms, and contribute to an increased appreciation of how effective cancer therapies are against background tumour growth. However, cancers are highly heterogeneous and differences in tumour growth rates across species and histiogenesis are expected to occur. Numerous studies and models have demonstrated high variability in tumour growth in response to treatment^2, 7, 25^. Likewise, an increasing number of statistical approaches in growth rate theory have attempted to capture the biological and mathematical complexities underlying growth rates^8, 26, 27^. Considering these complexities in neoplasms, understanding growth rates is essential to predict the effects of therapies. Though some types of cancers are caused by infectious agents such as viruses^28, 29^, only in rare circumstances do they become directly transmissible from one host to another. While there are only few cases known in nature: DFTD, the canine transmissible venereal tumour affecting domestic dogs^30^, and the recently found leukaemia type of cancer found in soft shelled clam^31^, there is also evidence that malignant sarcomas can be accidentally transplanted from patient to surgeon^32^ and from mother to foetus^33^. These cases highlight the capacity of cancer cells to escape their host and become transmissible. Given the nature of DFTD, a clonal transmissible cancer acting as an obligate parasitic cancer cell line, we acknowledge that caution should be taken when extrapolating our results to other cancers, in both humans and non-human animals. Nonetheless, our model based on empirical data from a wild population may be useful for parameterising mathematical models and further understanding growth rate theory in neoplasms.

Our devil—DFTD system is useful for studying tumour growth because individuals can be captured on multiple occasions and their tumours measured repeatedly and these wild animals are not subject to treatment. Furthermore, because DFTD is a clonal transmissible cancer of neuroendocrine origin, (and therefore all tumours have the same origin and there are minimal genetic differences between tumours), we can model growth rate in a system that allows capturing intrinsic growth properties over time. On one hand, this clonal cancerous cell-line of neuroendocrine origin allows minimising the numerous biological complexities behind growth rates in cancers from different origins. On the other hand, particular caution should be taken when comparing growth behaviour in other cancers in model organisms.

We acknowledge that substantive intraspecific variation in rates of tumour growth and carrying capacities may influence model parsimony. The differences we observed between individuals occurred irrespective of their sex. The statistical model used to fit the mathematical tumour growth models to the field data is necessarily exploratory due to the complex processes involved. A next step to clarify this complexity might involve a hierarchical Bayes approach, however, this would require a much larger dataset. Such a model could assume, for example, that the amount of time between tumour implantation and first observation follows a given continuous distribution. In particular, an Empirical Bayes approach could be used to estimate a prior for this distribution.

We recently demonstrated that lower infection rates and reduced population decline at our study site were associated with high prevalence of tetraploid tumours and that a sudden increase in diploid tumours was associated with higher infection rates and population decline^22^. This suggested that tetraploid tumours could have slower growth rates than diploid tumours, thereby offering a selective advantage by increasing the survival and reproductive output of infected individuals. These new results, however, tend to suggest the opposite effect. Diploids have a lower growth rate based on Bayesian credible intervals, though this difference was not statistically different based on a permutation test (see Table S1 and Fig. S1 in Supplementary Materials). Similarly, a higher growth rate in dermal tumours (outside of the oral cavity) was not replicated by a permutation test (Table S1). When the data were split between sexes, ploidy and location (Table S2), only the single *female-tetraploid-dermal* group showed any significant differences with other groups. This was due to a one - way effect (ploidy) as the only tetraploid group, with lack of other differences most likely due to low sample size. The lack of significant differences in growth rates between males and females (Table 2), tumour karyotypes and locations could suggest the need for a greater sample size to detect statistical significance, or that other mechanisms and adaptive forces, rather than growth rates, may explain the differences in the epidemiology and impact of the disease at the population level. These could be related to host immune status and tolerance to infection, genetic lineages of tumours not related to ploidy, or an interaction between host and tumour attributes.

Our framework allows fitting observable data from tumours of different karyotypes and locations to growth equations. Although we made simplifying assumptions to reflect the existing quantitative knowledge of tumour growth^10^, the results of our models can be extended to reflect more complex characteristics of growth behaviour in cancers. Further data on *in vivo* measurements of vascularization, metabolic and mitotic tumour rates (which are becoming increasingly feasible to obtain with advances in imaging technology) may help to capture the relationship between growth functions and cell biological parameters over time. Our models may offer insights into the natural patterns of tumour growth in the absence of any type of treatment, and therefore provide widely applicable base-line information for mechanistic tumour growth models to better understand the effects of cancer therapies. While our study provides insight into overall patterns of tumour growth, we acknowledge that other genetically controlled mechanisms such as apoptosis, autophagy or necrosis can also influence growth. Likewise, other intrinsic and extrinsic factors in our study population such as levels of stress in infected individuals, physiological and hormonal changes associated with reproduction that may result in immunosuppression and co-infection with other pathogens could also have an impact on tumour growth. Nevertheless, our study provides the first assessment of patterns of natural tumour growth, and therefore a basis from which to understand these additional complexities.

In conclusion, we have provided the first empirical estimates of tumour growth rates in a natural and untreated population of hosts. Our results may help to understand how tumours grow in other organisms, and more importantly, will help to parameterise numerical models of growth rates and predict potential effects of therapeutic treatment in solid tumours.

## Methods

### Field methods and data collection

Fieldwork and data collection were undertaken in a long-term monitoring site in north-western Tasmania (West Pencil Pine, see Hamede^22^) where the population has been sampled with a capture-mark-recapture framework at three month intervals since the beginning of the epidemic outbreak in 2006. Devils were trapped in custom made pipe traps (constructed from 30 cm diameter PVC pipe) and transferred into a hessian sack where they were handled, sexed and examined for DFTD status (see full details in section ‘Field protocols – handling procedures for data collection’ in Supplementary Materials). While being handled, the devil’s eyes were covered with the sack to calm the animal and allow examination. Wild Tasmanian devils have a freezing response to handling (unresponsive with no resistance to examination) and therefore no sedation is required. All fieldwork (with the exception of one trapping session) was undertaken by the same investigator (see Supplementary Material for further details). Tumours were measured using SPI precision callipers and tumour volume was estimated by converting maximum tumour length, width and depth of each tumour into cubic centimetres. Tumour karyotype (diploid, tetraploid – see refs 21, 22 for assessment of tumour karyotype) and the physical location of facial tumours (dermal – outside of the oral cavity; mucosal – inside of the oral cavity), to assess the potential effect of physical constraints on growth, were recorded. All methods for sampling animals, data collection and experimental protocols were approved by the University of Tasmania’s Animal Ethics Committee (approval permit number A0013326) and carried out in accordance with their guidelines and regulations.

## Tumour growth formulae

We use five models previously defined by Gerlee^10^ as potential candidates to describe growth in the volume of facial tumours over time. Each model was described by Gerlee^10^ as an initial value problem in terms of the differential equation that uniquely describes it. However, each is also analytically tractable, and both the differential equations and their solutions (given an initial condition *V* = *V*_0_ at *t* = *t*_0_) are given in Table 3, where *V* = volume in cm^3^ and *t* = time in days.

**Table 3:** Description of each of the growth models tested.

*Growth model*	*Differential equation*	*General solution*
*Exponential*	d*V*/d*t* = *rV*	*V* = *V*_0_ exp(*r*(*t* − *t*_0_))
*Logistic*	d*V*/d*t* = *rV*(1 − *V*/*K*)	*V* = *K*/[1 + (*K*/*V*_0_ − 1) exp(−*r*(*t* − *t*_0_))]
*Mendelsohn*	d*V*/d*t* = *rV* ^*b*^	*V* = ([1 − b] [*r*(*t* − *t*_0_) + *V*_0_^1−b^/(1 − b)])^1/(1−b)^
*Gompertz*	d*V*/d*t* = *rV* exp(−*ρ*(*t* − *t*_0_))	*V* = *V*_0_ exp(*r*/*ρ* [1 − exp(−*ρ*(*t* − *t*_0_))])
*von Bertalanffy*	d*V*/d*t* = *αV* ^2/3^ − *βV*	*V* = [α/β − (α/β − *V*_0_^1/3^) exp(−β(*t* − *t*_0_)/3)]^3^

Parameters *r* and α represent the tumour growth rate (per day), *K* the carrying capacity of the tumour, *b* an exponent determining the shape of the growth curve, *ρ* the proportional rate of decrease in growth rate, and *β* the rate of loss due to cell death (see Gerlee 2013 for details).

## Statistical model

We use a maximum-likelihood approach to fit our tumour data to each of the growth models, and Akaike information criteria corrected for small sample size (AICc) to select the most parsimonious model^34^. We assumed a lognormal error distribution in tumour volume to approximate measurement error, random variations in growth rate over time and between individual tumours. This approach also has the advantage that it is relatively computationally efficient and easy to implement. A particular issue with the available data is that it provides no context for the initial condition of each tumour. Though we know at what dates a particular tumour was measured and its volume at those dates, it was not possible to establish when it was first implanted in the individual and its initial volume – there is no consistent baseline across all tumours. We therefore include an extra parameter in the model for each tumour describing at which time *t*_0_ relative to its first observation that it reached an arbitrary small volume (*V*_0_ = 0.125 cm^3^), and optimise over this parameter in order to find the maximum likelihood for each tumour. The value of *V*_0_ is entirely cosmetic and has been set as an estimate of the minimum visible tumour size to provide reasonable visualisation of values for time since tumour detection (e.g. in Fig. 1). The only effect of changing this value is to shift all values of time *t* in a particular direction, (such that *V* = *V*_*0*_ when *t* = 0 on Fig. 1), but has no effect on our results. For example, a value for *V*_0_ of 100 cm^3^ would shift the range of *t* shown in the x-axis of Fig. 1 from approximately 0 to 600 days to approximately −400 to 200 days, but the shape of the fitted curve and its relationship with the data would be identical.

Where each tumour *i*, measured for the *j*th time, has volume *V*_*i,j*_ measured at time *t*_*i,j*_ relative to the first measurement (so *t*_*i,1*_ is always zero), is given an “initial” time *t*_*i*,0_ described above, and the given growth model *V*(*t*, {*θ, t*_*i*,0_}) is based on parameters *θ*, the likelihood of the parameters *θ* together with the initial times *t*_*i*,0_ for all tumours can be described as:

$${\rm{L}}(\theta ,{t}_{i,0},\varepsilon |{t}_{i,j},{V}_{{\rm{i}},{\rm{j}}})={{\rm{\Sigma }}}_{i,j}\,{\rm{N}}(\mathrm{log}({V}_{i,j});{\mu }=\,\mathrm{log}(V({t}_{i,j},\{\theta ,{t}_{i,0}\})),{\sigma }^{2}=\varepsilon )$$

where N(*x* | *µ*, *σ* ^2^) represents the probability density of the normal distribution with mean *µ* and variance *σ* ^2^ at value *x*, and *ε* represents the best-fit error rate. The maximum likelihood is then calculated by optimising over the free parameters *θ*, *t*_*i*,0_ and *ε* – the latter two can be optimised independently so dimensionality is not an issue.

## Electronic supplementary material

Supplementary Information
