# Supplementary material for: Untangling the model muddle: Empirical tumour growth in Tasmanian devil facial tumour disease
Source: Sci Rep. 2017 Jul 24;7:6217. doi: 10.1038/s41598-017-06166-3 (PMC5524923; doi:10.1038/s41598-017-06166-3)
Supplement: Supplementary file 1 — Supplementary Information [file 41598_2017_6166_MOESM1_ESM.pdf]

Supplementary Information

Untangling the model muddle: Empirical tumour growth in Tasmanian devil facial tumour disease

Rodrigo K Hamede, Nicholas J Beeton, Scott Carver and Menna E Jones

Table S1 Estimated ranges of maximum-likelihood parameters of logistic models based on a permutation test of 100,000 iterations, where karyotype and location are permuted randomly. Actual results from the main text (but with logistic model for mucosal) are given for comparison, with none of the actual parameter estimates outside of the central 95% interval of maximum-likelihood parameters based on randomly permuted data.

	r				*K* (cm^3)			
	2.5%	median	97.5%	**Actual**	2.5%	median	97.5%	**Actual**
Diploid	0.0114	0.0155	0.0243	**0.0163**	107.2	370.4	*Infinite*	**363.6**
Tetraploid	0.0079	0.0166	0.0319	**0.0260**	47.3	429.8	*Infinite*	**171.9**
Mucosal	0.0122	0.0155	0.0223	**0.0139**	142.3	363.5	*Infinite*	**337.6**
Dermal	0.0111	0.0155	0.0248	**0.0200**	101.5	374.5	*Infinite*	**226.3**

Table S2. Best fit and 95% central Bayesian credible intervals for tumour growth rate (r) and maximum tumour volume (K) using a logistic model in order to directly compare parameter values between sex, ploidy and location.

**Group**	**r**	**K**
Female, diploid, dermal	0.0156 (0.0103, 0.0248)	Inf (49.500, Inf)
Female, diploid, mucosal	0.0210 (0.0129, 0.0311)	59.470 (24.750, 247.500)
Female, tetraploid, dermal	0.0339 (0.0261, 0.0475)	137.412 (84.255, 220.000)
Male, diploid, dermal	0.0202 (0.0146, 0.0353)	293.649 (91.667, 990.000)
Male, diploid, mucosal	0.0123 (0.0070, 0.0179)	162.956 (57.558, 1650.000)

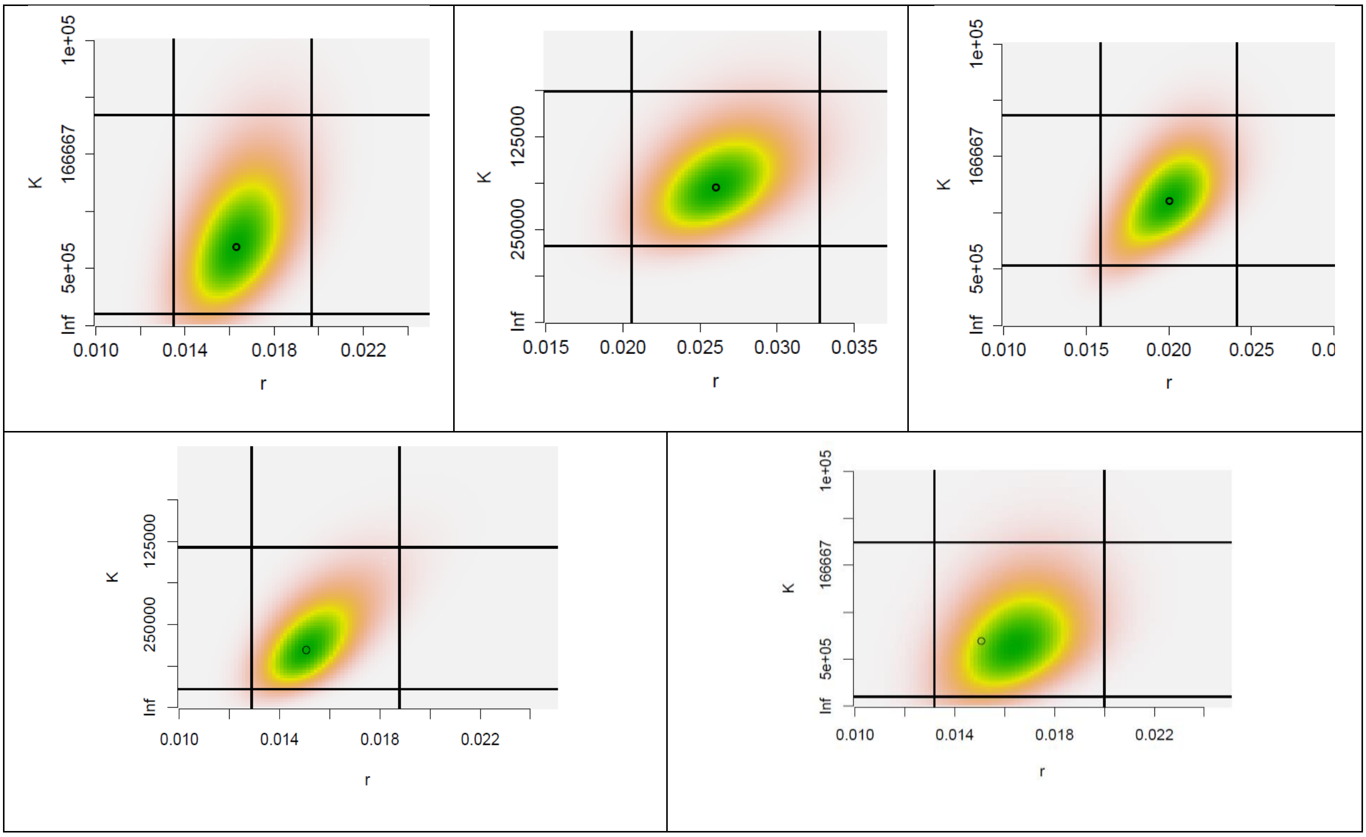

Figure S1 Plots of relative posterior probability for parameters in diploid (top-left), tetraploid (top-middle), dermal (top-right), female (bottom-left) and male (bottom-right) tumour models (note: results for mucosal tumours are not included, as the exponential model was selected), where green represents high probability. The maximum likelihood is denoted by a black circle, and the solid black lines represent Bayesian central 95% credible intervals given marginally for both parameters, based on independent uniform priors in *r* and the transformed variable *1/K* (corresponding to a prior distribution $p(K) = c/K^2$ for some constant *c*). As can be seen in the plots, these priors result in posterior surfaces that approximate a multivariate-normal distribution (elliptical in shape).

Field protocols – handling procedures for data collection

Handling procedures

Every captured animal is transferred by gently sliding it from the culvert pipe trap into a hessian sack and handled by the investigator. The animal's eyes are covered during the handling process to reduce stress. Devils are initially scanned for the presence of a microchip. If they are a new animal and not marked, a microchip (AllFlex©ISO FDX-B) is implanted subcutaneously between the shoulders at the nape of the neck. A series of standard morphometric measurements are recorded as well as size and location of all tumours if the animal has DFTD. Tumour measurements we were recorded using SPI precision callipers and converted to tumour volume by converting maximum length, width and depth into cubic centimetres. Handling procedures take on average 5-10 minutes depending on the number of tumours and biological samples to be collected. Because wild Tasmanian devils have a freezing response to handling (eg. they stay unresponsive and do not resist examination) it is possible to undertake all measurements, collection of data and biological samples without using any sedative. In addition, devils have high recapture rates (>80%) and because our study population has been visited at three month intervals since the beginning of the epidemic outbreak in May 2006, most of the individuals in our population are captured regularly throughout their life and are used to our handling procedures. The field investigator (R. Hamede) has more than 12 years of experience in handling Tasmanian devils and was responsible for all fieldwork throughout the study period with the exception of one trapping trip (November 2011). This trapping session was undertaken by an experienced research assistant (6 years of experience in the same study system). The research assistant was trained by R Hamede in the same study population.

All methods for sampling animals, data collection and experimental protocols were approved by the University of Tasmania's Animal Ethics Committee (approval permit number A0013326) and carried out in accordance with their guidelines and regulations.
